# Evidence for Reduced Drug Susceptibility without Emergence of Major Protease Mutations following Protease Inhibitor Monotherapy Failure in the SARA Trial

**DOI:** 10.1371/journal.pone.0137834

**Published:** 2015-09-18

**Authors:** Katherine A. Sutherland, Chris M. Parry, Adele McCormick, Anne Kapaata, Fred Lyagoba, Pontiano Kaleebu, Charles F. Gilks, Ruth Goodall, Moira Spyer, Cissy Kityo, Deenan Pillay, Ravindra K. Gupta, DART Virology Group

**Affiliations:** 1 University College London, London, United Kingdom; 2 Uganda Research Unit on AIDS, Medical Research Council (MRC), Uganda Virus Research Institute, Entebbe, Uganda; 3 School of Population Health, University of Queensland, Brisbane, Australia; 4 MRC Clinical Trials Unit at UCL, London, United Kingdom; 5 Joint Clinical Research Centre, Kampala, Uganda; 6 Wellcome Trust Africa Centre for Health and Population Sciences, University of KwaZulu Natal, Mtubatuba, South Africa; University of Rome Tor Vergata, ITALY

## Abstract

**Background:**

Major protease mutations are rarely observed following failure with protease inhibitors (PI), and other viral determinants of failure to PI are poorly understood. We therefore characterized Gag-Protease phenotypic susceptibility in subtype A and D viruses circulating in East Africa following viral rebound on PIs.

**Methods:**

Samples from baseline and treatment failure in patients enrolled in the second line LPV/r trial SARA underwent phenotypic susceptibility testing. Data were expressed as fold-change in susceptibility relative to a LPV-susceptible reference strain.

**Results:**

We cloned 48 Gag-Protease containing sequences from seven individuals and performed drug resistance phenotyping from pre-PI and treatment failure timepoints in seven patients. For the six patients where major protease inhibitor resistance mutations did not emerge, mean fold-change EC_50_ to LPV was 4.07 fold (95% CI, 2.08–6.07) at the pre-PI timepoint. Following viral failure the mean fold-change in EC_50_ to LPV was 4.25 fold (95% CI, 1.39–7.11, p = 0.91). All viruses remained susceptible to DRV. In our assay system, the major PI resistance mutation I84V, which emerged in one individual, conferred a 10.5-fold reduction in LPV susceptibility. One of the six patients exhibited a significant reduction in susceptibility between pre-PI and failure timepoints (from 4.7 fold to 9.6 fold) in the absence of known major mutations in protease, but associated with changes in Gag: V7I, G49D, R69Q, A120D, Q127K, N375S and I462S. Phylogenetic analysis provided evidence of the emergence of genetically distinct viruses at the time of treatment failure, indicating ongoing viral evolution in Gag-protease under PI pressure.

**Conclusions:**

Here we observe in one patient the development of significantly reduced susceptibility conferred by changes in Gag which may have contributed to treatment failure on a protease inhibitor containing regimen. Further phenotype-genotype studies are required to elucidate genetic determinants of protease inhibitor failure in those who fail without traditional resistance mutations whilst PI use is being scaled up globally.

## Introduction

It is estimated that almost 15 million HIV-infected people in resource limited settings are currently being treated with antiretroviral therapy[1]. Most will have started ART with a PI-sparing regimen, as recommended by WHO guidelines[2], leaving the PI class available for use in combination second-line therapy. The use of boosted protease inhibitor monotherapy (bPImono) as maintenance therapy has been investigated in a number of trials in resource-rich settings, which have suggested that this strategy can be considered under certain circumstances[3–7]. The Boosted Protease Inhibitor Monotherapy as Maintenance Second-line Anti-retroviral therapy in Africa (SARA) trial, was a nested pilot study within the DART trial[8] designed to test whether ritonavir boosted lopinavir (LPV/r) monotherapy (bPImono) after an initial 24 weeks on second-line combination therapy provided similar outcomes to continuation on combination second-line therapy (CT) [9]. 192 patients who had experienced clinical/immunological failure on first-line therapy in the DART trial [8] and had received 24 weeks of second-line LPV/r containing therapy were randomized and the trial demonstrated non-inferiority of LPV/r monotherapy in CD4+ T cell response and rate of serious adverse events (SAEs). However, viremia (≥50 copies/mL) was more common 24 weeks after randomization in the bPImono arm (23% CT vs 40% bPImono, p = 0.01). Major resistance mutations in protease [10] were detected in 5/20 (25%) bPImono participants with a VL>1000 copies/ml at 24 weeks/last time-point with successful genotyping (compared to 0/8 CT)[9]. More recently, the larger EARNEST trial (also conducted in sub Saharan Africa) showed inferiority of a PI monotherapy approach to triple therapy over 2 years of follow up [11], though detailed genotypic resistance data have not been published.

Clinical studies of PI-based combination regimens have previously highlighted our poor understanding of determinants of virological failure to this drug class when assessed by *protease* genotyping alone [12–15]. *Protease* genotyping and phenotyping remains the standard in commercial systems and clinical trial settings. By contrast, in research settings, mutations in *gag* have been shown to affect PI susceptibility directly and have been associated with treatment failure where accompanied by major protease mutations (as reviewed by Fun et al. [16]). Inclusion of co-evolved Gag alongside protease in phenotypic assays enables more accurate measurement of PI susceptibility than protease alone [17]. Recently we noted lower inherent PI susceptibility of subtype AG and G viruses (which circulate mainly in West Africa) as compared to subtype B viruses (known to predominate in western Europe and North America), using a full-length *gag-protease* assay [18]. Furthermore, studies have described mutations in Gag in subtype C viruses associated with PI and exposure and treatment failure but their direct effect on susceptibility was not reported [19, 20] Given that *gag* is polymorphic between HIV-1 subtypes, assessments of PI susceptibility using such assays are warranted across regions and subtypes in the context of PI exposure.

We set out to investigate PI susceptibility of full-length Gag-Protease containing viruses, derived from SARA trial participants harbouring HIV-1 subtypes A and D from Uganda. The ability to perform genotypic and phenotypic resistance testing before PI exposure and after PI-containing therapy failure offered a unique opportunity to explore the determinants of treatment failure in viruses circulating in East Africa, where an estimated 4.6 million people are HIV infected (http://www.unaids.org/en/regionscountries/countries).

## Methods

### Patients

Seven patients who experienced treatment failure in the SARA trial were studied and the pre-PI and treatment failure timepoints samples assessed genotypically and phenotypically for each patient. For this study, patients were considered to have failed therapy if they had a viral load above 1,000 copies/mL at week 24 of the SARA trial. In SARA, viral load monitoring and resistance testing were performed retrospectively and did not inform treatment decisions. Pre-PI therapy was defined as the time at which the patient switched to second-line LPV/r containing combination therapy (24 weeks before randomization in SARA)[9]. Four patients were selected from the 20 SARA participants randomized to bPImono experiencing virological failure (defined as VL>1000 copies/ml at week 24 and/or last visit) with successful protease resistance genoptyping[9], based on the availability of sample or population sequence covering full-length Gag-Protease from both pre-PI therapy and failure time-points–patients 2, 3, 4 and 6 (Sutherland et al, in press AIDS Research and Human Retroviruses). Two of the eight patients experiencing failure in the CT arm, patients 1 and 7 were also included. Patients with major resistance mutations pre-PI therapy or at failure were excluded from our study cohort. However, patient 5 who failed with major resistance mutation I54V that was not present before therapy was examined separately to assess the fold-change in PI susceptibility in our system conferred by a known major PI resistance mutation. This facilitated comparison of fold-changes in susceptibility with other studies, and put into context the magnitude of susceptibility changes observed within the present study. All clinical data were collected as part of the SARA clinical trial. Data quality control was carried out in line with principles of good clinical practice.

### Ethics statement

Written informed consent for participation was obtained from participants. SARA received ethics approval in Uganda (Uganda Virus Research Institute (UVRI) Science and Ethics Committee) and the UK (Imperial College). SARA is registered under ISRCTN53817258.

### Genotypic testing methods

Standard genotypic resistance testing by RT-PCR covering protease was performed as previously described [21]. In addition, Gag-Protease was amplified for sequencing using one step cDNA synthesis and first round PCR with primers ATT GTG TGA CTC TGG TAA CTA GAG ATC CCT (HXB2 nucleotides 570–599) and antisense TCC TAA TTG AAC YTC CCR AAR TCY TGA GT (HXB2 2799–2828), and second round PCR with primers TCT CTA GCA GTG GCG CCC GAA CAG (HXB2 626–649) and GGC CAT TGT TTA ACY TTT GGD CCA TCC. (HXB2 2597–2623) Viral subtype of Gag-Protease was determined using REGA HIV-1 Subtyping tool[22]. Sequences were manually checked and edited using DNADynamo software.

### Cloning of patient derived Gag-Protease

For patients with available extracted RNA after routine viral load and genotypic resistance testing, full-length *gag-protease* was amplified by cDNA synthesis and nested PCR as previously described [23]. Clonal analysis was performed for at least six viral variants at each timepoint and the variant most similar to the population sequence selected for phenotyping. Sequences were analysed in DNADynamo and manually examined for mixed bases and errors such as stop codons or frameshift mutations. When RNA extract was not available, but genotyping of *gag* and *pol* had been successfully performed, the population *gag-protease* sequence was synthesized (GenScript, USA). Synthesis was performed for patient 2, 4 and 7 failure timepoints, and patient 6 pre-PI and failure timepoints. Full-length *gag-protease* was cloned into the Gag-Pol expression vector p8.9NSX+ using unique NotI and XhoI restriction sites as previously described [23, 24].

### PI susceptibility and single-round infectivity

PI susceptibility and single-round infectivity were measured using cell-based, single replication-cycle phenotypic assays, as previously described [24]. Briefly, 293T cells were co-transfected with the Gag-Pol expression vector, VSV-g envelope vector and luciferase reporter vector and incubated with serial dilutions of PI 18 hours post-transfection to measure susceptibility. Infectivity was measured in fresh 293T cells using SteadyGlo luciferase substrate (Promega) and EC_50_ calculated using logistic regression, expressed as a fold-change compared with the p8.9NSX+ reference strain. Susceptibility was measured to the PIs lopinavir (LPV) and darunavir (DRV), obtained from the NIH AIDS Reagent Program. Single-round infectivity was measured by infection of 293T cells in the absence of drug, with virus produced by transfection of 293Ts as described above and harvested 48 hours post-transfection. PI susceptibility and single round infectivity data presented here are means of at least two independent experiments. Two-tailed, paired t-tests were performed, with P > 0.05 considered statistically significant, using Graph Pad PRISM 5 software (La Jolla, CA, USA).

### Phylogenetic analysis

Phylogenetic analyses were carried out for patients where multiple clonal sequences were available at both timepoints. Nucleotide sequences from pre-PI therapy and treatment failure timepoints of Gag-Protease were aligned in MEGA6 software [25] using the ClustalW algorithm and phylogeny construction performed in PHYLIP (http://evolution.genetics.washington.edu/phylip.html) using the maximum likelihood method under the generalized time reversible (GTR) model. Trees were constructed with 500 bootstrap replicates and viewed using FigTree v1.3.1 software (http://tree.bio.edu.ac.uk/software/figtree/) and MEGA6 software.

## Results

**Fig 1:**
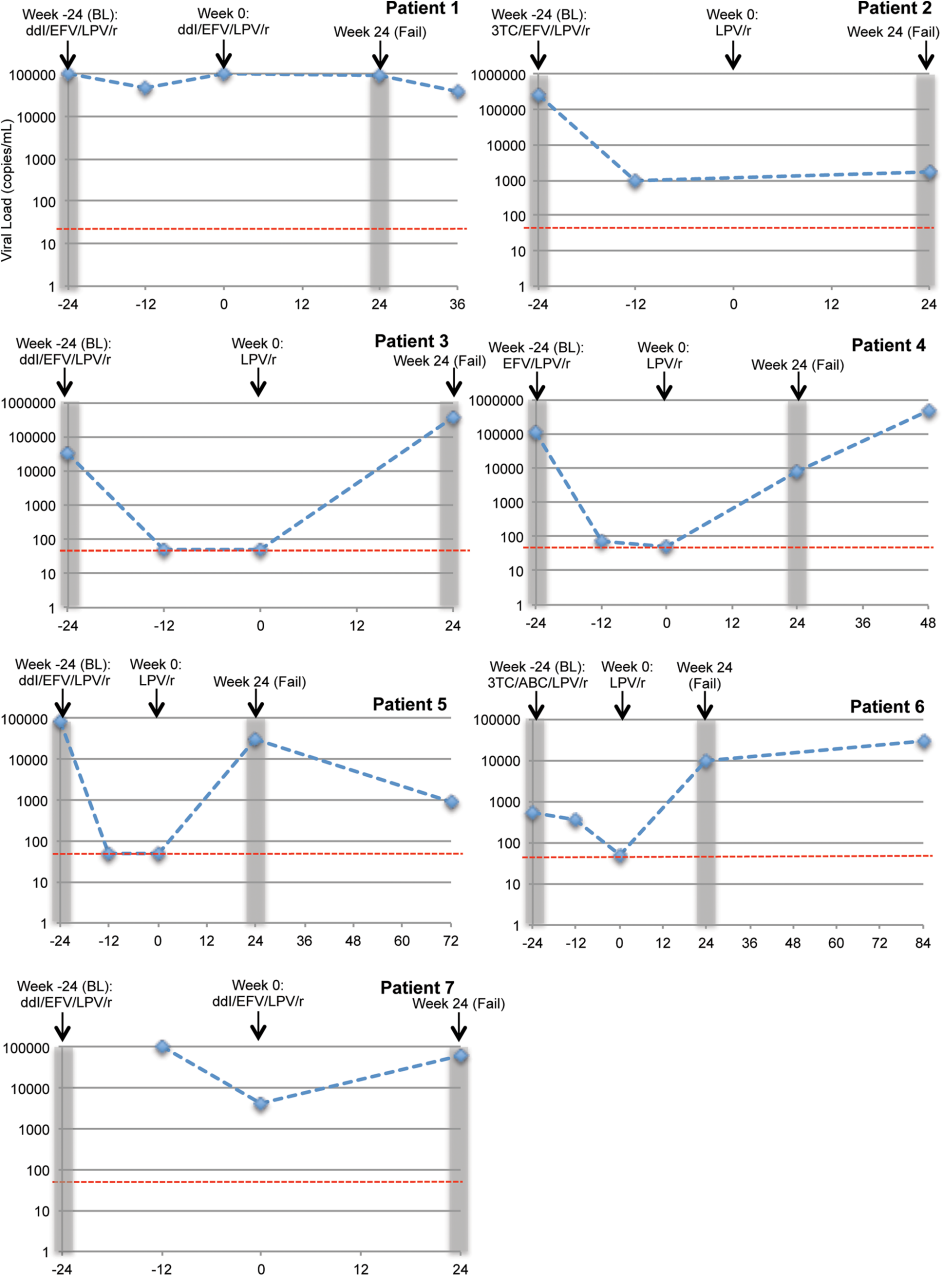
Viral load and patient treatment information. Treatment history of each patient is shown, from switch to second-line therapy during the DART trial (week -24, BL) and their enrollment in SARA (week 0), shown for most patients by simplification to LPV/r monotherapy. All patients were randomised to the LPV/r monotherapy arm of the SARA trial except for patients 1 and 7, who were randomised to continue on LPV/r containing triple therapy (CT). Available viral load measurements are shown; these were performed retrospectively and did not inform treatment decisions. The limit of detection for the assay (<50 copies/mL) is denoted with a red dashed line. The time from which the pre-PI (baseline, BL) and failure (Fail) samples included in this study were derived is highlighted by grey bars. For patient 7, the pre-PI sample was derived from week 0 of the DART trial before the week -24 of SARA timepoint, hence is not shown on this graph.

To explore the determinants of treatment failure, we investigated whether reduced susceptibility before PI therapy, or the development of reduced PI susceptibility during therapy in the absence of major resistance mutations, may have contributed to treatment failure in this population of treatment-experienced patients. Viral load trajectories and treatment histories of the six participants are shown in Fig 1.

Three patients were infected with subtype A (patient 5, 6 & 7) and four with subtype D (patient 1–4) by protease sequencing performed as part of standard resistance testing during the trial[9] Following our full-length *gag-pro* sequencing we established that two patients initially classified as being infected with subtype D viruses were in fact infected with a recombinant form comprising a stretch in matrix from subtype A with the rest of *gag-pro* being subtype D (patients 1 and 4). All four individuals in the bPImono arm for whom a viral load measurement was available at the time of simplification to monotherapy (after 24 weeks of PI-containing combination therapy, patients 3, 4, 5 and 6), had a viral load below limit of quantification (<50 copies/ml), including patient 5 who failed with major resistance mutations, demonstrating adequate adherence and drug potency at this early time point. Interestingly, patient 2 did demonstrate a significant reduction in viral load after switch to PI containing therapy, although no measurement at week 0 was available and experienced failure with a comparatively low viral load– 1164 copies/mL. Most patients were switched from a first-line regimen of ZDV/3TC/TDF to second-line regimens containing efavirenz (EFV) and either didanosine (ddI) or lamivudine (3TC) with ritonavir boosted lopinavir (LPV/r). However, patient 4 was switched to a two drug regimen comprising only EFV and LPV/r (Fig 1). Of note, patients 1 and 7 did not achieve viral load suppression after switch to LPV/r containing second-line therapy despite randomization to continuation on combination therapy in SARA, which could suggest poor adherence to treatment (Fig 1). Both developed the K103N major NNRTI resistance mutation conferring high level resistance to EFV at failure, and in addition patient 1 had mutations predictive of intermediate level resistance to ddI pre-PI and at failure.

Comparison of Gag-protease sequences derived from pre-PI and failure timepoints was performed for each patient and amino acid changes present at failure in comparison with the pre-PI timepoint noted (Table 1). In patients 1 and 3, only two amino acid changes were present at failure, but in the remaining patients numerous mutations were present (Table 1). However, no pattern of mutations was present across patients. Patient 5 developed major resistance mutations I54V and I84V in protease at failure, along with 31 other mutations including A431V and P453L in Gag that have been previously associated with PI exposure and resistance[16].

**Table 1:** Amino acid changes in *Gag-protease* at time of treatment failure relative to pre-PI Gag-Protease sequence in study participants.

	Mutations at Treatment Failure, not present at the Pre-PI Timepoint	
Patient	Gag	Protease
1	R26K, G387S	-
2	L46I, R91G, A95K, T109A, S111F, N124S, S239T, R268K	V13I
3	V34I	I77V
4	L34I, A37P, D42E, Q59M, K69R, L90E, T122A, N389I, Q468E, V469I, P473S, T487A	I13V, V72I
5	K26R, R30Q, E62A, T72S, Y79F, A81T, V82T, H89Q, R91Q, I94V, N124S, K127T, T138A, M142W, T144H, V147L, V215I, S252G, T332S, K335R, T339A, R406K, A431V*, P453L, L462I, G466R, S472F, N479S, E482D	I54V*, R57K, P63L, I84V*
6	V7I, G49D, R69Q, A120D, Q127K, N375S, I462S	-
7	S38G, A67T, N127K, K361R, P473A	-

*I54V and I84V were not present in the same viral clone, but were present on separate clones. A431V developed only in the clones with I54V. ‘-‘) denotes when no changes in protease were present at failure.

**Fig 2:**
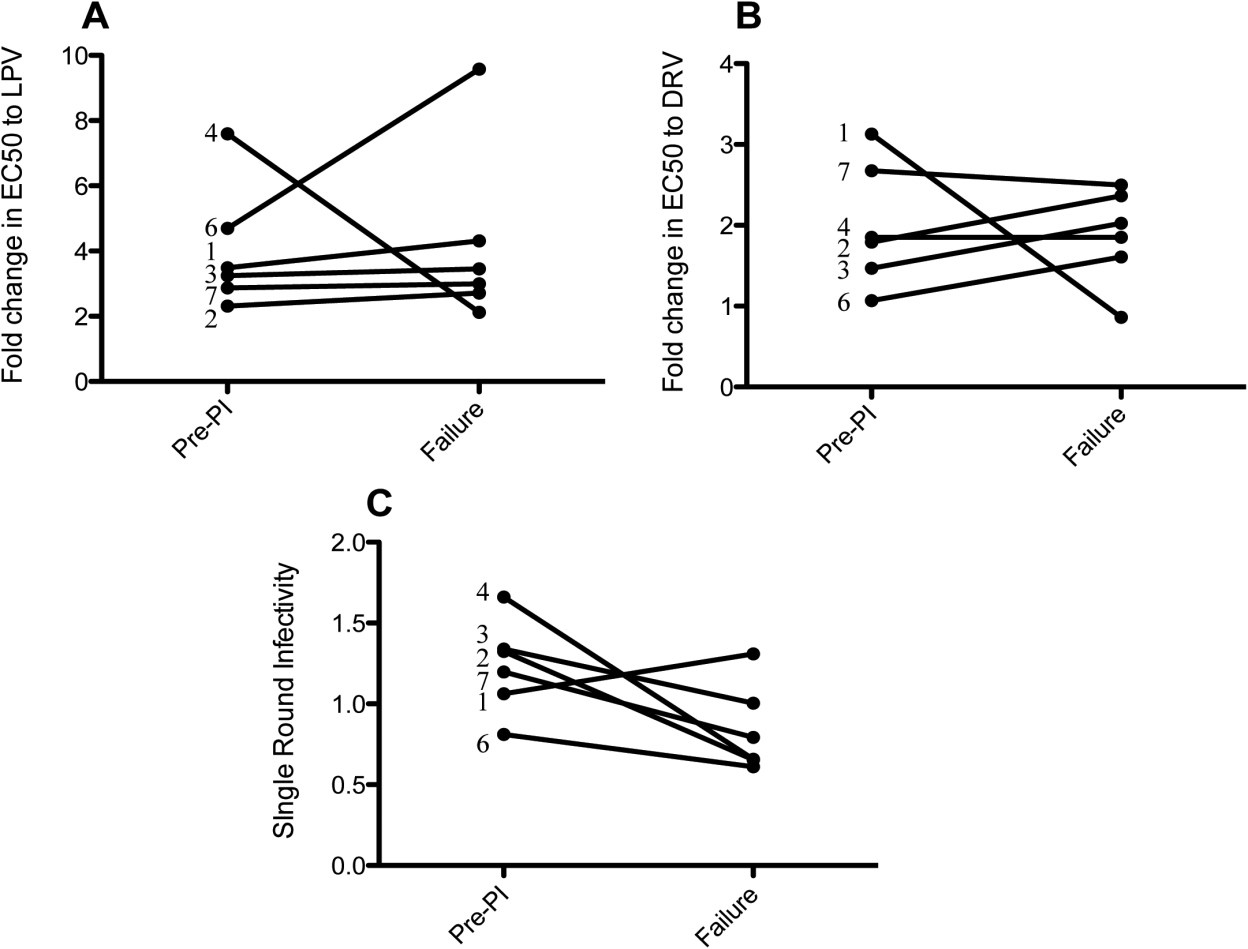
PI susceptibility and single round infectivity of patient Gag-Protease derived pre-PI therapy and at the time of treatment failure. Full-length Gag-Protease from pre-PI therapy and failure time-points was amplified or synthesised, and cloned into our Gag-Pol expression vector p8.9NSX+. PI susceptibility of VSV-g pseudotyped viruses from patients experiencing virological failure in the absence of major resistance mutations was measured in a cell-based, single-round, phenotypic assay to the PIs A) Lopinavir (LPV) and B) Darunavir (DRV). The patient numbers are shown for each data point and data are means of two independent repeats. Our data demonstrate no significant difference in susceptibility between pre-PI therapy and failure viruses to the PIs LPV (t test, p = 0.91) and DRV (t test, p = 0.78), in patients failing in the absence of major resistance mutations. (C) Single-round infectivity of viruses was measured in the absence of drug in HEK 293T cells and compared in patients failing without major resistance mutations. No significant decrease in single-round infectivity was present (t test, p = 0.07).

Susceptibility to LPV, the PI used as treatment in the study, and the second generation PI DRV (sometimes available as third line/salvage therapy in resource limited settings) was measured using an established phenotypic drug resistance assay. We tested viruses present in patients pre-PI therapy (at the time of switch to second-line LPV/r containing combination therapy—24 weeks before SARA enrollment) and at failure (at least 24 weeks after SARA enrollment and randomization)—the timing of the samples in context with viral load and trial week is shown Fig 1. Fig 2 shows the fold-change in PI susceptibility for pre-PI and treatment failure viruses from each patient experiencing failure without major resistance mutations (patients 1–4, 6 & 7) to the PIs LPV and DRV. For viruses derived from the pre-PI time-point, the mean fold-change in EC_50_ to the PI LPV was 4.07 fold (95% CI, 2.08–6.07) relative to the clade B assay reference strain and 2.00 fold (95% CI, 1.19–2.80) to DRV (Fig 2). At failure the mean fold-change in EC_50_ to LPV and DRV was 4.50 fold (95% CI, 0.82–8.19) and 1.71 fold (95% CI, 0.97–2.46), respectively. There was no statistically significant change in susceptibility between pre-PI and failure time points for either LPV (Fig 2A, p = 0.91) or DRV (Fig 2B, p = 0.78). In an analysis of replication capacity, there was no significant change in single-round infectivity between pre-PI therapy and failure viruses (p = 0.07) (Fig 2C).

**Fig 3:**
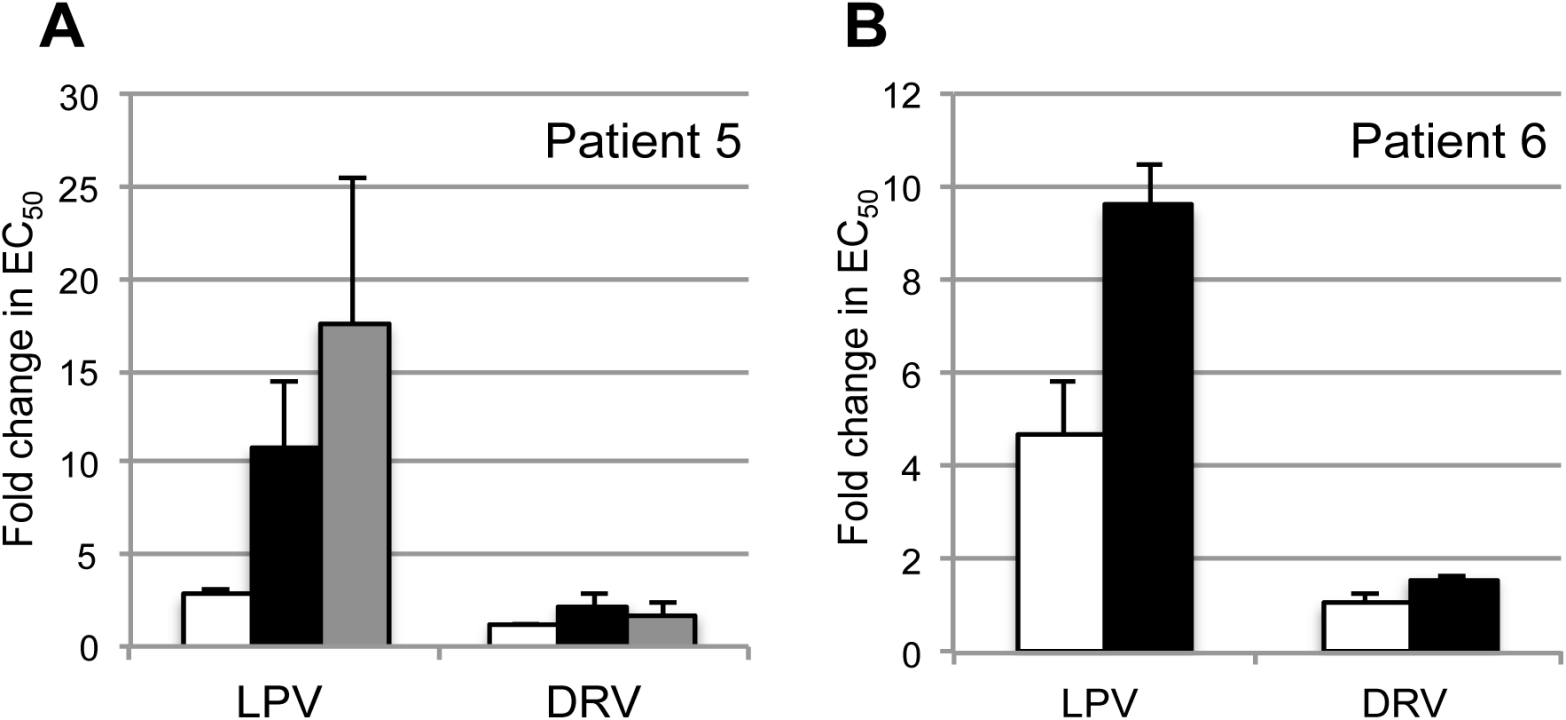
PI susceptibilities of pre-PI and failure virus from two patients demonstrate changes in phenotypic susceptibility over time. Full-length Gag-Protease sequences from pre-PI therapy and failure time-points was amplified or synthesised, and single round phenotypic susceptibility testing performed. PIs tested were Lopinavir (LPV) and Darunavir (DRV). (A) Susceptibility data from patient 5 demonstrates the reduction in susceptibility conferred by major mutations I84V (black) and I54V (grey) in our system in comparison with the patient virus at pre-PI therapy (white). Error bars represent standard deviation of two independent experimental repeats. (B) Virus derived pre-PI therapy (white bar) and at time of failure (black bar) from patient 6 demonstrated a difference in susceptibility to LPV in the absence of the development of major resistance mutations.

Patient 5 was included to measure the effect of major resistance mutations in a subtype specific background in our phenotypic assay. Population genotypic resistance testing had identified the I54V resistance mutation alongside the A431V compensatory Gag mutation at the time of failure [26]. Our clonal analysis also identified the I84V major resistance mutation present in two of seven failure variants in the absence of A431V or I54V. Both the I54V/A431V and I84V variants tested separately displayed 10–17 fold reductions in susceptibility in comparison with the clade B reference strain, illustrating the level of reduced susceptibility that a single major protease resistance mutations confer in our assay system (Fig 3A). Comparison of EC_50_ values between the pre-PI and failure timepoint showed a 3.28 and 3.62 fold reduction in susceptibility conferred by the development of the major mutations I84V and I54V respectively (S1 Table).

Phenotyping data revealed that patient 6 was of particular interest. Patient 6 was infected with a subtype A virus and experienced virological failure on LPV/r monotherapy in the absence of major PI resistance mutations. We detected a significant decrease in susceptibility to LPV from 4.7 fold pre-PI to 9.6 fold at the time of treatment failure in the absence of major resistance mutations or any other mutations in protease between the pre-PI and failure timepoints, correlating with a 2-fold increase in LPV EC_50_ (Fig 3B and S1 Table). This reduction in susceptibility did however correlate with the appearance of a number of amino acid changes in Gag (V7I, G49D, R69Q, A120D, Q127K, N375S and I462S). One of these, N375S, is located in the p2/NC cleavage site and has been previously reported to be associated with PI exposure in vivo[27, 28].

**Fig 4:**
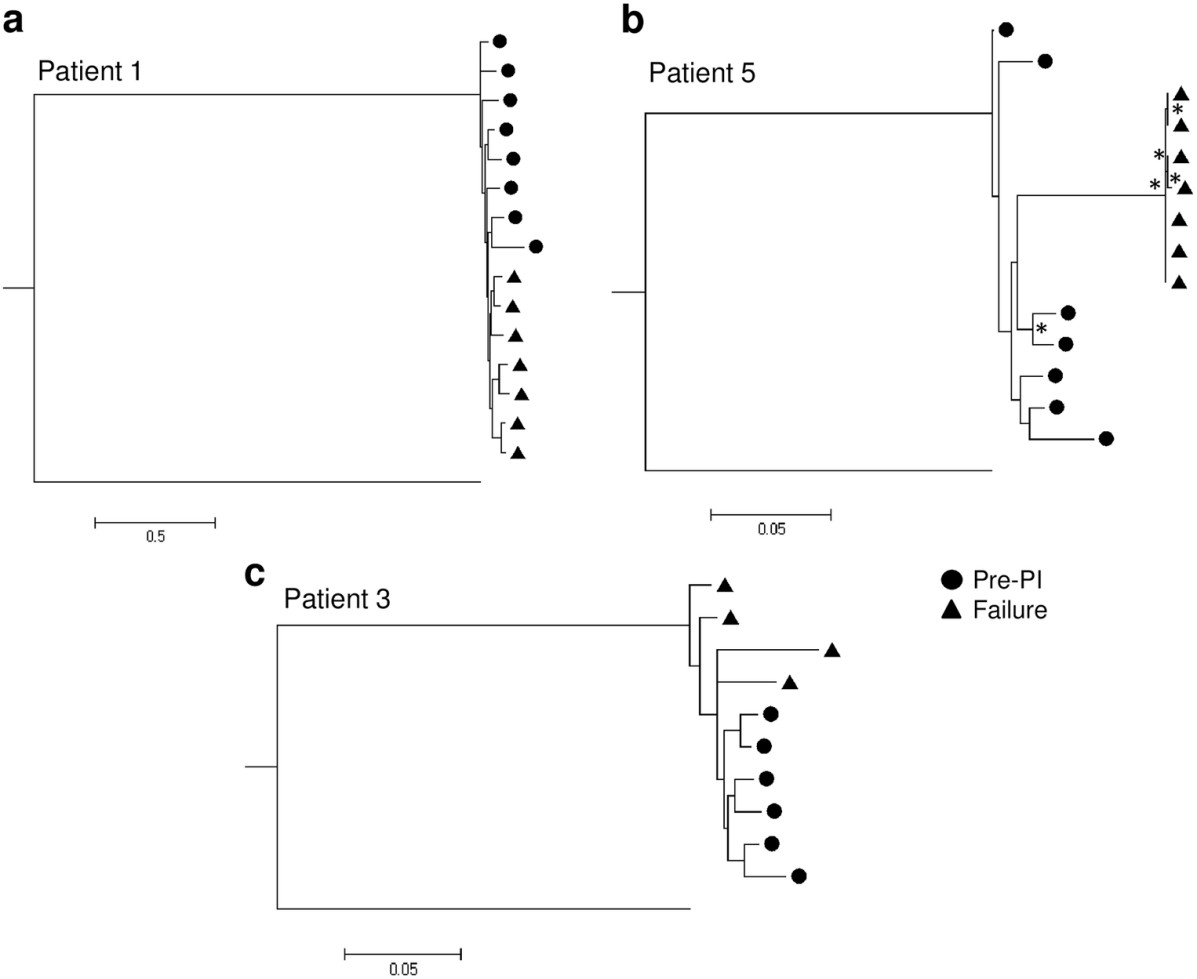
Maximum Likelihood phylogeny of virus from pre-PI therapy and treatment failure timepoints. Phylogenetic reconstruction was performed using the GTR model of nucleotide substitution. Phylogeny for the three patients for whom multiple sequences from both baseline and failure timepoints is shown: (a) patient 1, (b) patient 5 and (c) patient 3. Pre-PI virus sequences are denoted with circles and failure virus sequences with triangles. For patient 5 the drug resistance positions Gag 431 and Protease 54 and 84 were stripped from the alignment before phylogenetic re-construction. Nodes separating pre-PI and failure variants that supported by >75% bootstrapping are depicted with an asterisk (*).

For patients where sample availability enabled clonal analysis at both pre-PI therapy and failure time-points, phylogenetic reconstruction of the available sequences was performed to examine the genetic relationship of the viruses before and after PI exposure. In each case, the phylogenetic trees provide evidence for the emergence of a genetically distinct viral population at the time of treatment failure in comparison with pre-PI therapy, indicating ongoing viral replication and evolution during LPV/r monotherapy (Fig 4). For patients 1 and 3, a clear separation of pre-PI therapy and failure variants was present, but this separation was not statistically supported–most likely due to the close relatedness of intrapatient viral variants (Fig 4A and 4C). Separation was particularly evident, and supported statistically, in patient 5 (Fig 4B) where the failure viruses formed a distinct cluster with a comparatively long branch length. Of note, Fig 4B was constructed using an alignment stripped of resistance positions 431 in Gag and 54 and 84 in protease, indicating that the evolution driven by PI exposure is not restricted to the classically described resistance positions. Phylogenetic reconstruction performed with these resistance positions resulted in an identical tree (data not shown).

## Discussion

The global scale-up of antiretroviral therapy has resulted in increasing numbers of patients experiencing virologic failure and qualifying under WHO guidelines for switch to PI-containing second-line therapy (mostly LPV/r due to availability of a heat stable co-formulation of lopinavir and ritonavir). Of those experiencing PI failure, only a minority have resistance detectable by standard approaches to drug resistance testing. As may be expected studies have linked suboptimal PI concentrations with poorer therapy outcomes [29, 30], but there remains a need for greater understanding of the determinants of PI therapy failure as these drugs become more widely used in populations infected with divergent HIV-1 strains. We have therefore applied a Gag-Protease phenotypic susceptibility assay to six patients failing PI-based therapy in the SARA trial who had no detectable protease resistance mutations, and one patient who did develop major protease resistance mutations.

Overall, amongst the six patients failing without major protease mutations we observed no significant difference in PI susceptibility between pre-PI therapy and failure viruses, indicating that treatment failure on LPV/r is unlikely to be solely attributable to the development of reduced susceptibility under PI pressure (Fig 2). This is in agreement with findings for patients enrolled in the French MONARK PI monotherapy clinical trial [31]. Furthermore, viruses present pre-PI therapy were relatively susceptible to LPV displaying fold-changes in susceptibility to LPV below 5 (mean fold-change 4.07). In this cohort, there was no evidence that reduced susceptibility at baseline may have contributed to treatment failure which contrasts with our findings in subtype CRF02_AG viruses from patients failing PI in MONARK that displayed mean baseline fold change of 10.4 at baseline (17).

A single patient (6) displayed a significant reduction in susceptibility at failure in comparison with pre-PI therapy viruses, accompanied by amino acid changes in Gag only. These changes in Gag conveyed a similar reduction in LPV susceptibility to the major PI resistance mutations I54V or I84V present singly at failure for patient 5 (Fig 3). This observation is reminiscent of the MONARK resistance analysis, where one in four developed reduced PI susceptibility during therapy, correlating with the emergence of three changes in Gag as well as the secondary mutation M36I in protease [31]. These observations are in-keeping with other studies demonstrating that amino acid positions in Gag can directly affect susceptibility to protease inhibitors, independent of compensating for reduced fitness following emergence of major resistance mutations [32–35]. Taken together, these studies indicate that the development of reduced susceptibility during PI therapy in the absence of protease resistance mutations may contribute to treatment failure in a subset of patients and further exploration of the genetic determinants in Gag is required. It is likely that this reduced susceptibility may render the patient more likely to experience treatment failure when therapy adherence is suboptimal.

In the remaining patients the causes of treatment failure on PI regimens remain unknown. It is likely that poor adherence may have contributed to treatment failure in some patients, but in the absence of plasma drug concentrations the exact role is unknown. For patients 1 and 7 randomised to the continuation arm it is clear that the development of K103N, a major NNRTI resistance mutation conferring high-level resistance to EFV, may have contributed to treatment failure. However, given that susceptibility to PIs was maintained it could be argued that the patients were effectively receiving PI monotherapy, as the RTIs in their regimen were compromised by resistance, and that viral suppression should still have been maintained. Further research to explore the determinants of treatment failure in patients receiving PI-containing combination therapies is urgently required, given that these are the regimens most frequently utilised in resource limited settings.

Of note, we frequently found evidence of ongoing viral evolution during LPV/r monotherapy using phylogenetic analysis, despite observing no change in phenotypic susceptibility. This evolution could be impacting susceptibility in ways that are not assessed in our assay, for example involving coevolution with envelope mutations as suggested by Rabi et al. [36], or other routes which could be addressed in the future using patient derived full-length viruses. Other limitations of this study include the sample size, largely driven by sample availability and amplification efficiency, and sometimes necessitating the use of commercial gene synthesis. Patient Gag-Protease was assessed in a subtype B backbone and it is possible that this may have affected replication fitness, although this backbone has also been used with subtype C, AG and G viruses[17, 31]. We hypothesise that the increase in susceptibility at failure observed here for patient 4, which is puzzling and not in-keeping with the rest of our patient observations, could be a result of coevolution of Gag-Protease with regions outside those included in our assay. It is also possible that the synthesis of a population sequence at the failure timepoint, rather than clonal analysis, has resulted in the inclusion of mutations that would not actually be found on the same genomes within the patient and are therefore not naturally coevolved.

Although small sample size is a limitation of this study, paired samples taken before treatment and at failure are difficult to obtain and very little data on PI susceptibility of Gag-Protease in non-B subtypes within a clinical setting exists. To date a single study has examined PI susceptibility of Gag-Protease from subtype A viruses, demonstrating the value of including Gag in phenotypic analysis, but this study did not include paired samples taken pre- and post-therapy[17]. To our knowledge there are no data for subtype D clinical isolates. Here, the identification of recombinants precluded assessment of impact of subtype on drug susceptibility.

In conclusion, we have performed the first detailed phenotypic analysis of PI-therapy failure using co-evolved Gag-Protease in viruses that predominate in East Africa. We provide further evidence that the development of reduced PI susceptibility during LPV/r based treatment in the absence of major resistance mutations, albeit in only a single patient and we hypothesise that this may contribute to treatment failure in a small subset of patients. In these and other patients failing PI, suboptimal adherence is likely to be a key contributory factor. Importantly, our study also suggests that in patients experiencing relatively early failure on LPV, viruses remain fully susceptible to DRV- a drug that may be used as a salvage therapy option for these patients in the future.

## Supporting Information

S1 Table Pre-PI and failure timepoint EC_50_ values for LPV and DRV. (DOCX)

## Abbreviations

HIV: human immunodeficiency virus
LPV: lopinavir
LPV/r: ritonavir boosted lopinavir
DRV: darunavir, PI–protease inhibitor
Gag: Group antigen, Pro–protease
SARA: The Boosted Protease Inhibitor Monotherapy as Maintenance Second-line Anti-retroviral therapy in Africa
RTI: reverse transcriptase inhibitor
