# Correction: Evidence for Reduced Drug Susceptibility without Emergence of Major Protease Mutations following Protease Inhibitor Monotherapy Failure in the SARA Trial

**DOI:** 10.1371/journal.pone.0157094

**Published:** 2016-06-02

**Authors:** Katherine A. Sutherland, Chris M. Parry, Adele McCormick, Anne Kapaata, Fred Lyagoba, Pontiano Kaleebu, Charles F. Gilks, Ruth Goodall, Moira Spyer, Cissy Kityo, Deenan Pillay, Ravindra K. Gupta, DART Virology Group

The title of this article should include “HIV-1.” The correct title is: Evidence for Reduced HIV-1 Drug Susceptibility without Emergence of Major Protease Mutations following Protease Inhibitor Monotherapy Failure in the SARA Trial. The correct citation is: Sutherland KA, Parry CM, McCormick A, Kapaata A, Lyagoba F, Kaleebu P, et al. (2015) Evidence for Reduced HIV-1 Drug Susceptibility without Emergence of Major Protease Mutations following Protease Inhibitor Monotherapy Failure in the SARA Trial. PLoS ONE 10(9): e0137834. doi:10.1371/journal.pone.0137834.
