# Supplementary material for: Evidence for Reduced Drug Susceptibility without Emergence of Major Protease Mutations following Protease Inhibitor Monotherapy Failure in the SARA Trial
Source: PLoS One. 2015 Sep 18;10(9):e0137834. doi: 10.1371/journal.pone.0137834 (PMC4575205; doi:10.1371/journal.pone.0137834)
Supplement: S1 Table — (DOCX) [file pone.0137834.s001.docx]

| Patient | LPV EC_50_ | | DRV EC_50_ | |
| --- | --- | --- | --- | --- |
|  | Pre-PI | Fail (fold^a^) | Pre-PI | Failure (fold) |
| 1 | 21.7 | 28.9 (1.33) | 11.2 | 7.0 (0.63) |
| 2 | 8.9 | 10.4 (1.17) | 7.2 | 9.4 (1.31) |
| 3 | 8.9 | 9.8 (1.1) | 5.9 | 8.1 (1.37) |
| 4 | 37.3 | 10.7 (0.29) | 11.0 | 5.4 (0.49) |
| 5 (to I84V) | 26.6 | 87.3 (3.28) | 1.2 | 2.3 (1.91) |
| (to I54V) |  | 96.3 (3.62) |  | 1.8 (1.5) |
| 6 | 17.7 | 35.5 (2.0) | 6.5 | 9.7 (1.49) |
| 7 | 6.6 | 7.0 (1.06) | 3.7 | 3.4 (0.92) |

**S1 Table. Pre-PI and failure timepoint EC_50_ values for LPV and DRV**

^a^ Fold is the fold difference in EC50 at failure in comparison with the pre-PI timepoint.
